# Ceftazidime for Neutropenic Fevers: Is It Still an Appropriate Choice?

**DOI:** 10.6004/jadpro.2013.4.6.2

**Published:** 2013-11-01

**Authors:** Ellen Bethany Napier

**Affiliations:** From Jefferson University Physicians, Philadelphia, Pennsylvania

## Abstract

Infection continues to rank as a primary cause of treatment-related mortality in patients with cancer. For patients with neutropenic fevers, immediate empiric treatment with antibiotics is standard care. However, which specific antibiotic is best for initial treatment of high-risk patients has been much debated without consensus. Many major health centers use intravenous ceftazidime as first-line therapy for these patients. Yet updates to guidelines published by the Infectious Diseases Society of America and the National Comprehensive Cancer Network suggest that ceftazidime may no longer be an optimal choice. This article reviews the literature regarding ceftazidime therapy for the treatment of high-risk neutropenic patients with fevers. This critical analysis of existing research reveals significant pharmacologic, physiologic, social, and financial implications, and recommendations for further studies are made.

Infection continues to rank as a primary cause of treatment-related mortality in patients with cancer. Fever may be the first measurable sign of infection; it is also a common finding in patients whose immune systems are rebuilding. Thus, the appropriate agent for the treatment of neutropenic fevers must cover potential infections without causing unnecessary toxicity or resistance. Patients with prolonged neutropenia or any significant comorbidities are considered to be at high risk; see Table 1 (Robbins, 2011). When a high-risk neutropenic patient is febrile, immediate empiric treatment with antibiotics is standard care. There has been much debate surrounding the specific antibiotic that is optimal for the initial treatment of these patients, yet no consensus has been reached.

**Table 1:**
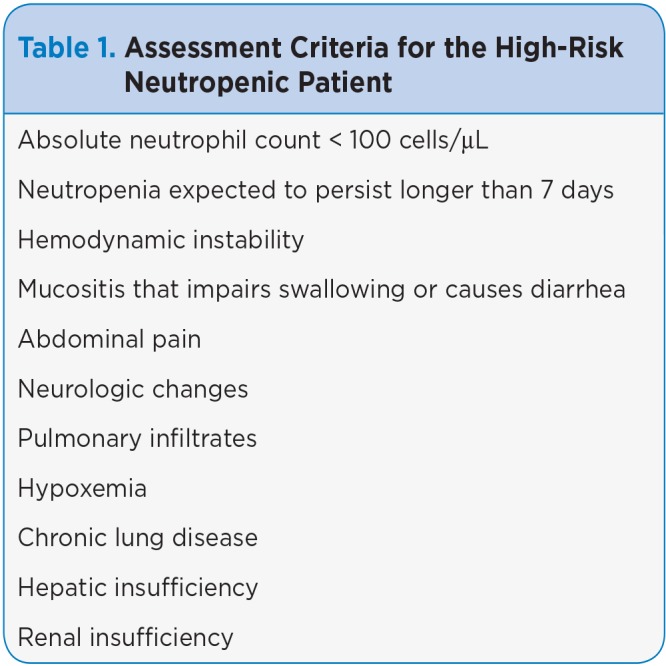
Table 1. Assessment Criteria for the High-Risk Neutropenic Patient

Many major health centers use IV ceftazidime as first-line therapy for these patients. Yet updates to published guidelines by the Infectious Diseases Society of America (IDSA, 2011) and the National Comprehensive Cancer Network (NCCN, 2013) suggest that treatment with ceftazidime may no longer be the best course of action. The following is a review of the literature surrounding ceftazidime use in this patient population, along with an exploration of the pharmacologic, physiologic, social, and financial implications of high-risk febrile neutropenia.

## Background and Significance

Since the 1970s, when a landmark study in The New England Journal of Medicine demonstrated that prompt empiric treatment with broad-spectrum antibiotics significantly reduces morbidity and mortality (Schimpff, Satterlee, Young, & Serpick, 1971), immediate treatment of neutropenic fevers became standard care. At that time, there was a predominance of Gram-negative bacterial infections in neutropenic patients, but through the 1980s and 1990s Gram-positive bacteria became the more common infecting organisms. This shift was largely due to the increased use of indwelling plastic venous catheters, which allow a point of entry for Gram-positive skin flora (Freifeld et al., 2011). Research during the 1980s and 1990s centered on monotherapy vs. combination antibiotic therapy, and monotherapy was found to be just as effective as combination therapy with an aminoglycoside, with less toxicity (Paul, Yahav, Bivas, Fraser, & Leibovici, 2010).

Today over 80% of patients with hematologic malignancies being treated with chemotherapy and 10% to 50% of patients with solid tumors will have fever and neutropenia (Freifeld et al., 2011). Infection is a potentially life-threatening complication of cancer therapy that must be treated as a medical emergency (Robbins, 2011). Organizations such as the IDSA and the NCCN make detailed recommendations for the care of febrile neutropenic patients, specifying algorithms for prophylaxis, risk stratification, diagnostic testing, and treatment (Freifeld et al., 2011; NCCN, 2013). However, the optimal choice of initial antibiotic therapy for high-risk patients continues to be in question.

## Physiologic Principles

Neutrophils are a subset of leukocytes (see Table 2), and neutropenia may be only one aspect of a patient’s myelosuppression. But as the most numerous leukocytes and the body’s primary phagocytic agents, neutrophils are "the first line of defense" and play a critical role in protecting the body from foreign antigens, including bacteria. In addition, neutrophils have a profoundly rapid life cycle: They are made in the bone marrow at a rate of approximately 80 million per minute, living only 2 to 3 days, compared to a macrophage, which typically lives months (Yarbro, Wujcik, & Gobel, 2010). This makes them particularly at risk during chemo- and radiotherapies, treatments that target rapidly dividing cells. The singular importance and extreme fragility of neutrophils makes them a critical prognostic indicator: An absolute neutrophil count (ANC) of less than 1,000 cells/ìL has historically been identified as a threshold for high risk of infection (Gobel, Triest-Robertson, & Vogel, 2009).

**Table 2:**
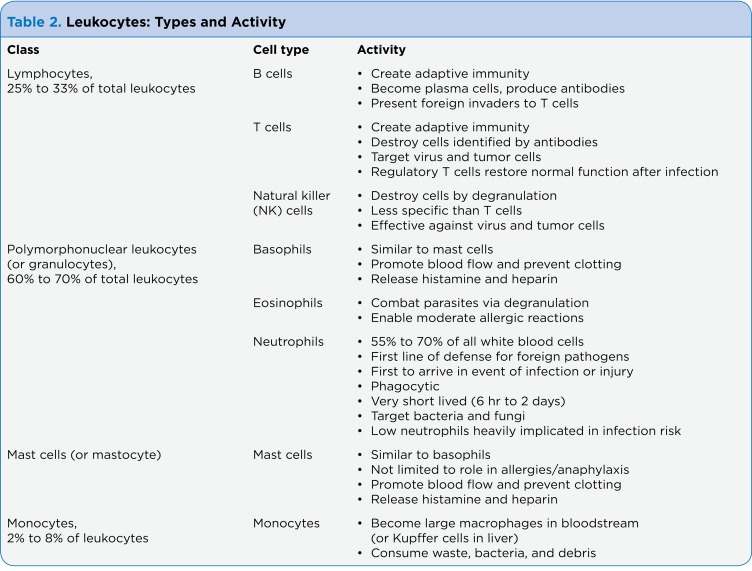
Table 2. Leukocytes: Types and Activity

Additionally, in the absence of neutrophils, the body may not be able to mount the expected reaction to an infection, causing muted or absent symptoms (such as not producing the sputum which signals a respiratory infection). As such, it is critical to monitor fever as an early (though nonspecific) sign. Fever in the context of neutropenia warrants immediate intervention.

Patients with febrile neutropenia may be considered low risk if they have solid tumor malignancies, have an anticipated neutropenic period of less than 7 days, and live within 1 hour of a hospital. These patients may be managed on an outpatient basis and may receive oral antibiotic treatment (Robbins, 2011).

Patients are considered high risk if they have any of the following conditions: ANC < 100 cells/ìL, neutropenia expected to persist longer than 7 days, hemodynamic instability, mucositis that impairs swallowing or causes diarrhea, abdominal pain, neurologic changes, pulmonary infiltrates, hypoxemia, chronic lung disease, and/or hepatic or renal insufficiency (see Table 1). These patients need to be admitted to a hospital urgently and given immediate empiric IV antibiotics.

## Published Guidelines

For high-risk neutropenic fevers, current IDSA guidelines recommend "monotherapy with an anti-pseudomonal beta-lactam agent, such as cefepime, a carbapenem (meropenem or imipenem/cilastatin), or piperacillin/tazobactam" (Freifeld et al., 2011, p. e57). While the 1997 IDSA guidelines recommended ceftazidime specifically by name (Hughes et al., 1997), the 2010 updates deleted this listing. In the 2010 updates, Freifeld and colleagues go on to say, "Many centers have found that ceftazidime is no longer a reliable agent for empirical monotherapy of fever and neutropenia because of its decreasing potency against Gram-negative organisms and its poor activity against many Gram-positive pathogens, such as streptococci" (Freifeld et al., 2011, p. e67).

The NCCN recommends monotherapy with one of the following agents: imipenem/cilastatin, meropenem, piperacillin/tazobactam, cefepime, or ceftazidime (NCCN, 2013). However, the NCCN guidelines give ceftazidime a 2B rating (appropriate, based on lower-level evidence), while the other listed agents are rated category 1 (appropriate, based on higher-level evidence). The NCCN also included a footnote to ceftazidime reading, "weak Gram-positive coverage and increased breakthrough infections limit utility" (NCCN, 2013, p. FEV-5). While ceftazidime is still considered appropriate therapy for febrile neutropenic patients, is there evidence in the literature that suggests other antibiotics could provide better outcomes?

## Review of the Literature

To review the literature, various databases were searched in November 2011, including CINAHL and Ovid MEDLINE. Studies cited in relevant articles and significant reviews were also explored. Only English language articles were considered. Studies limited to pediatric patients were excluded.

From this literature search, the eight existing studies were identified, seven of which explore ceftazidime for the treatment of febrile neutropenic patients. Six studies compared drug efficacy, and two examined resistance patterns. In addition, the search revealed one meta-analysis of beta-lactams for empiric treatment of neutropenic fevers. When the results of these studies are considered as a group, ceftazidime has been studied directly in comparison to the four other drugs recommended by the IDSA and the NCCN: cefepime, imipenem, meropenem, and piperacillin/tazobactam. A discussion of each comparison follows, with a summary presented in the Figure. Further information on the individual drugs is provided in Table 3.

**Figure 1:**
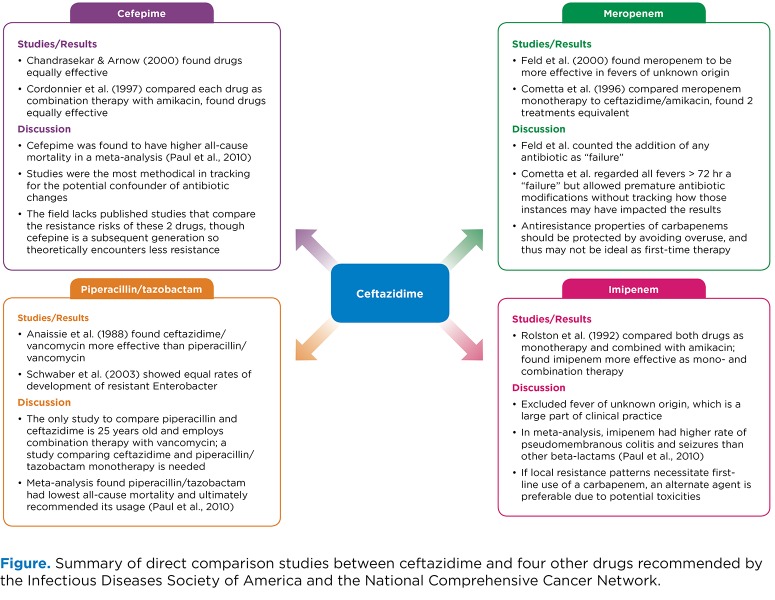
Figure. Summary of direct comparison studies between ceftazidime and four other drugs recommended by the Infectious Diseases Society of America and the National Comprehensive Cancer Network.

**Table 3:**
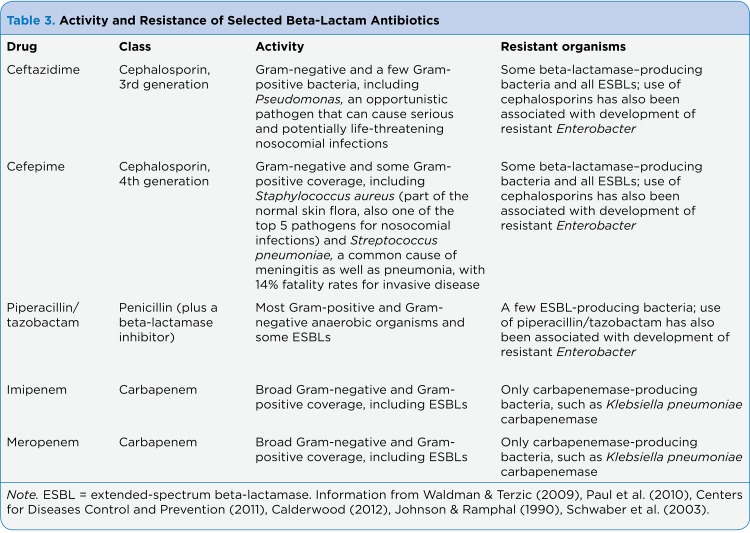
Table 3. Activity and Resistance of Selected Beta-Lactam Antibiotics

**CEFEPIME**

In two direct comparison studies (Chandrasekar & Arnow, 2000; Cordonnier et al., 1997), ceftazidime was found to be statistically equivalent in efficacy to cefepime, with and without the addition of amikacin, even though cefepime provides broader Gram-positive coverage. There have been no studies directly comparing resistance patterns between these two drugs, though cefepime is a subsequent generation of cephalosporin and so theoretically is less susceptible to certain beta-lactamases (inactivating enzymes) produced by bacteria.

In a meta-analysis of beta-lactams for neutropenic fevers by Paul et al. (2010), cefepime was found to have higher all-cause mortality than other beta-lactams. The US Food and Drug Administration (FDA) refuted this claim, though the FDA’s analysis (1) used over 30 unpublished trials by Bristol-Myers Squibb, the company that produces cefepime and (2) included studies where cefepime was used in combination therapy with aminoglycosides (Kim et al., 2010). The FDA’s conclusions about the safety of cefepime monotherapy were based on data from combination therapy and privately funded, uncorroborated, unpublished trials. As such, Paul and colleagues maintain that published data suggest cefepime bears a higher rate of all-cause mortality for this specific patient population, though this finding has never been adequately explained. Therefore, evidence suggests that ceftazidime appears to be a better choice in comparison to cefepime.

**PIPERACILLIN**

The only published English language study to compare piperacillin and ceftazidime appeared in 1988, when combination therapy was still thought to be necessary. This study by Anaissie et al. compared ceftazidime/vancomycin with piperacillin/vancomycin, with a third study arm in which patients received all three antibiotics (piperacillin, ceftazidime, and vancomycin). Ceftazidime/vancomycin was found to be more effective than piperacillin/vancomycin. However, it is not possible to surmise the comparative effectiveness of monotherapy from this study. In addition, the study data were collected over 25 years ago. Since then, infection patterns have changed, so questions of efficacy need to be reexamined. A study by Schwaber, Graham, Sands, Gold, and Carmeli (2003) found ceftazidime and piperacillin/tazobactam to have equivalent risks for developing resistant Enterobacter.

The meta-analysis by Paul et al. (2010) found that piperacillin/tazobactam had the lowest all-cause mortality relative to the other beta-lactams, and in the end recommended its usage for empiric treatment of neutropenic fever. However, the data on which this meta-analysis is based include studies such as those by Anaissie et al. (1988). It seems reasonable to conduct head-to-head studies between piperacillin/tazobactam and ceftazidime before concluding the superiority of the piperacillin regimen.

**IMIPENEM**

In a single study, ceftazidime was found to be less effective than imipenem, with and without amikacin (Rolston et al., 1992). However, this study excluded fevers of unknown origin, which make up a significant portion of clinical practice: An infection source is isolated in approximately 30% of neutropenic fevers (Robbins, 2011). Additionally, compared to the other antibiotics of interest, imipenem has also been associated with higher rates of pseudomembranous colitis and seizures (Feld, DePauw, Berman, Keating, & Ho, 2000). It is difficult to deduce the superiority of either drug until a study can be conducted that does not disregard fevers of unknown origin. It is well documented that carbapenems evoke far less bacterial resistance than cephalosporins. As these antiresistance properties should be aggressively protected by avoiding overuse, carbapenems may not be ideal as first-line therapy. However, even if a carbapenem agent is necessitated by local resistance patterns, an alternate carbapenem may be preferable due to potential toxicities.

**MEROPENEM**

In studies that compare meropenem with ceftazidime, one trial found meropenem to be significantly more effective, particularly when used in hematopoietic stem cell transplant (HSCT) patients (Feld et al., 2000), while another trial found meropenem to be equivalent to ceftazidime/amikacin (Cometta et al., 1996). It is interesting to note that the advantage of meropenem in the Feld et al. study seems to come entirely from fevers of unknown origin; ceftazidime and meropenem were equivalently effective in treating both clinically and microbiologically defined infections. However, both of these studies had problematic measures of "success" and "failure" with relation to the addition of other antibiotics, an issue that merits further discussion.

## Analysis of the Literature

Within all of the comparative efficacy studies, overall success rates for ceftazidime in the treatment of neutropenic fevers ranged from 21% to 60% (and up to 71% when combined with amikacin). This wide range in efficacy points to one of the difficulties in comparing data across studies: The measures of "clinical failure" and "success" were difficult to define and employ uniformly. This was particularly problematic as it related to the issue of modification of antibiotic regimens.

In the six efficacy studies, "success" was generally defined as the resolution of fever and any clinical signs of infection and the eradication of any identified infecting organism (some studies also specified that patient must remain afebrile for at least 4 to 7 days). However, the studies differed widely in how they treated the modification of regimens and use of additional antibiotics (e.g., cases where a practitioner prescribed vancomycin in addition to the antibiotic being studied). For example, in comparing meropenem to ceftazidime, Feld et al. (2000) categorized cases as "failures" with the addition of any antibiotic, no matter when or why it occurred, and changes could be made at the investigator’s discretion. Cometta et al. (1996) seemed the least precise on the issue, regarding all fevers persisting 72 hours a "failure," but allowing premature antibiotic modifications without tracking how those instances may have impacted the results.

The most methodical studies included those by Chandrasekar & Arnow (2000) and Cordonnier et al. (1997) comparing cefepime and ceftazidime, which allowed the addition of vancomycin but tracked the use and impact of the drug and found it was equivalent in both arms of the study. Rolston et al. (1992) allowed the addition of amikacin and built it into the study structure (comparing both ceftazidime and imipenem as monotherapy and in combination with amikacin).

A more precise and measurable standard of efficacy or success is 30-day all-cause mortality. This is the primary measure that Paul and colleagues (2010) used in their meta-analysis of beta-lactams for use in neutropenic fevers. It is easily measurable and can be applied across studies without the need for clinician interpretation.

In future studies, changes in antibiotic regimens could be controlled by allowing changes to the antibiotic regimen in accordance with NCCN guidelines. For example, the NCCN (2013) recommends specific alterations in the antibiotic regimen if particular localized symptoms of infection are present (such as abdominal pain or vesicular lesions) and identifies situations in which the addition of vancomycin is indicated. In future studies, cases where treatment differs from the NCCN guidelines may be discarded, rather than being counted as regimen "failures." Cases where health-care providers added additional antibiotics in accordance with the NCCN guidelines could be tracked for the various study arms and analyzed as a secondary outcome in the final results. Other secondary outcomes that were not addressed in the previous studies but should be considered include length of stay and days in an ICU setting.

In the end, the existing data leave several opportunities for further study. The comparable efficacy of cefepime and ceftazidime seems reliably documented, due to the sound design and implementation by Chandrasekar and Arnow (2000) and Cordonnier et al. (1997), though the potential greater all-cause mortality of cefepime suggests ceftazidime is a superior choice. Existing studies comparing ceftazidime to carbapenems were problematic, and it is important to preserve carbapenems’ superiority against resistant organisms by not using them as first-line treatment unless absolutely necessary. Studies comparing ceftazidime to piperacillin/tazobactam as monotherapy do not yet exist yet would seem to be most useful at this point.

## Organizational and Financial Implications

Hospitalizations required for high-risk patients with febrile neutropenia are associated with significant mortality, morbidity, and financial cost. In 2002, the direct cost associated with treating cancers was estimated at $60.9 billion (Caggiano, Weiss, Rickert, & Linde-Zwirble, 2005), with hospital costs accounting for an estimated 40% to 50% of the cost of total cancer care (Kuderer, Dale, Crawford, Cosler, & Lyman, 2006). A study performed from a consortium of 115 academic health centers and teaching hospitals found that these institutions alone spent $1.06 billion in 6 years on hospitalizations for febrile neutropenic patients, even excluding the significant costs for patients who had undergone HSCT.

Furthermore, 1 in 14 of these patients hospitalized for neutropenic fevers died (Caggiano et al., 2005). High-risk patients accounted for 74% of the overall hospital days, 78% of the hospital costs, and 64% of the inpatient deaths (Kuderer et al., 2006). Current modes of management continue to encumber individuals, families, and health-care organizations with substantial mortality and financial cost. Improvements provide a substantial opportunity to save lives and resources.

## Human Diversity, Ethics, and Social Implications

There are no studies of neutropenic fevers in racial minorities or in patients with various educational levels and/or from differing socioeconomic backgrounds. Further, none of the eight existing studies reviewed in this paper considers or mentions race or socioeconomic status at all. Only one study, Feld et al. (2000), includes race (using the categories "White" and "Other") in the demographic analysis of the study groups, but does not mention it in the analysis or discussion.

By ignoring the potential impact of race or socioeconomic status on study results, the authors of the various studies are in essence denying that it is a factor in the problem of interest. Yet the statistics demonstrate definitively that race and socioeconomic status play a substantial role in cancer treatment and outcomes. Cancer death rates for those with the least education are almost double those for the most educated individuals. African American men have a 33% higher death rate from cancer than white men, while African American women have a 17% higher death rate from cancer than white women (Siegel, Ward, Brawley, & Jemal, 2011). If the goal of these studies is to prevent premature cancer deaths, the authors have missed a very important opportunity to add to our collective pool of data and knowledge.

## Summary and Future Directions

Despite advances in management over the past 40 years, febrile neutropenia continues to burden individuals, our health-care system, and our society. High-risk neutropenic patients consume the most resources and have the greatest mortality rates. Determining which antibiotic provides the best outcome for these patients could make a profound impact.

A review of existing literature reveals that opportunities exist to expand our knowledge. Updated studies are needed to evaluate the comparable efficacy of ceftazidime relative to piperacillin/tazobactam. New studies should employ 30-day all-cause mortality as a primary measure and allow for the addition of other antibiotics according to NCCN guidelines. Secondary measures should include modifications to the treatment regimen, ethnicity/race, socioeconomic indicators (such as level of education), length of stay, and days in an ICU bed. In addition, more studies need to be done in HSCT patients, whose neutropenia and clinical course vary more than those of a solid tumor patient who is considered high risk due to, for example, hepatic or renal insufficiency.

Though these studies would require an investment of resources, they have the potential to reduce the great cost, both in terms of finances and mortality, commanded by febrile neutropenia today. Advanced practitioners in oncology are uniquely positioned to play a leadership role in this endeavor, applying their high-level and cost-effective skills to clinical trials, national and institutional policy reviews, and direct patient care.
